# Query-based-learning mortality-related decoders for the developed island economy

**DOI:** 10.1038/s41598-022-04855-2

**Published:** 2022-01-19

**Authors:** Chien-Hung Yeh, Yining Wang, Fu-Chun Yeh

**Affiliations:** 1 School of Information and Electronics, Beijing Institute of Technology, Beijing, 100081 China; 2 Nuffield Department of Clinical Neurosciences, University of Oxford, Oxford, OX3 9DU UK; 3 Department of Aeronautical and Opto-Mechatronic Engineering, Vanung University, Taoyuan, 320 Taiwan

**Keywords:** Health care, Risk factors, Mathematics and computing

## Abstract

Search volumes from Google Trends over clear-defined temporal and spatial scales were reported beneficial in predicting influenza or disease outbreak. Recent studies showed Wiener Model shares merits of interpretability, implementation, and adaptation to nonlinear fluctuation in terms of real-time decoding. Previous work reported Google Trends effectively predicts death-related trends for the continent economy, yet whether it applies to the island economy is unclear. To this end, a framework of the mortality-related model for a developed island economy Taiwan was built based on potential death causes from Google Trends, aiming to provide new insights into death-related online search behavior at a population level. Our results showed estimated trends based on the Wiener model significantly correlated to actual trends, outperformed those with multiple linear regression and seasonal autoregressive integrated moving average. Meanwhile, apart from that involved all possible features, two other sets of feature selecting strategies were proposed to optimize pre-trained models, either by weights or waveform periodicity of features, resulting in estimated death-related dynamics along with spectrums of risk factors. In general, high-weight features were beneficial to both “die” and “death”, whereas features that possessed clear periodic patterns contributed more to “death”. Of note, normalization before modeling improved decoding performances.

## Introduction

The outbreak of potentially fatal diseases, especially those communicable, raised public high attention, particularly whilst suffering from the prolonged coronavirus epidemic^1^. A timely and accurate quantitative nowcast of disease-driven possible mortality supports the government to take preventive health steps against the outbreak of diseases, whilst a more comprehensive understanding of the regularities on the potentially fatal diseases supports formulating healthcare policies. For example, a recent multinational, prospective cohort study showed varying associations for the 14 potentially modifiable risk factors (e.g., behavioral, metabolic, socioeconomic, and psychosocial factors, etc.) with mortality and cardiovascular disease over 150 thousand participants from 21 countries^2^. For a possible death trend nowcast technology to be developed for widespread use beyond sophisticated calibrations, the decoder must remain stable over a long period, and the conversion of the inputs to the estimated trend must be robust, stable, and reliable. The amount of information captured depends on the number of states that can be decoded, the accuracy of decoding, and the speed and/or latency at which this decoding occurs.

Traditionally, estimation of mortality trend was performed per medical records, the hysteresis nature of medical records plus the immense workload in collections and analyses of statistical data both limit its practical use nevertheless. To investigate the hidden regularities of nature, big data mining has gradually broadened as a potential approach^3,4^, of which the online trace reports facilitate surveillance for disease transmission in particular. At first, the wiki-based data-logs were utilized to predict disease spreads in some countries^5,6^, nevertheless, the text-formatted data-logs confront the difficulties in revealing fine-scale temporal and spatial representations (i.e., less than a country), thus limiting the pursuit for a finer location and/or the exploration for a specific period^5^. Later, search queries from Google include Google Dengue Trends and Google Flu Trends were accessed for infectious disease outbreak forecasting^7–9^ yet terminated services in 2015; follow on this, keyword search volumes over temporal databases from Google Trends became publicly available. Google Trends, unlike the wiki-based data-logs, enables finer spatial segmentations with clearer-defined temporal scales, was successively implemented to predict seasonal influenza and/or dengue fever in several countries^10–12^.

More researches based on Google Trends have been reported in the last decade^10–13^, with its applications into a wide range of fields including tourism, elections, communications, business, and economics, especially in the fields of health and medicine^14^, e.g. recent studies have reported that Google queries effectively in monitoring the suicide rates^15,16^. The emphasis of the related studies had gradually shifted from describing and diagnosing the trends toward predicting and nowcasting the occurrence of outbreaks, as well as forecasting seasonal diseases’ prevalence. Recent studies employed Google data have displayed promising results in predicting various diseases and outbreaks, e.g., AIDS^17^, influenza-like illness^18^, and suicide risk^15^, etc. Methods include support vector regression^19^, autoregressive-integrated moving average model^20^, ensemble methods^21^, phenomenological models^22^, and deep learning algorithm^23^, etc., which may integrate with signal processing technique and/or optimization algorithm, were applied to trend predictions; e.g., Fahad Shabbir Ahmad et al. predicted mortality in paralytic ileus patients using electronic health records with a hybrid machine learning framework^24^.

Our previous findings revealed that both high-weight and annual periodic patterns contribute to the prediction of death-related queries for the continent's economy^11^. However, whether or not similar models and/or features fit well to an island economy remained unclear. Taiwan, as a representative developed island economy with a relatively homogeneous healthcare system, serves as an ideal model to explore its mortality levels along with the changes of the death cause spectrum, of which the latter is essential to guide preventative response strategies^6^. On the other side, concerning the traditional mortality surveillance system may require 1–2 weeks to aggregate and process the data, thus the up-to-date search queries volumes obtained from Google Trends enable the estimates to be consistently 1–2 weeks ahead of the government reports. To this end, we constructed and compared the mortality-related decoders based on the Wiener model, the linear regression model, as well as the Seasonal Autoregressive Integrated Moving Average model (SARIMA) in this work, of which the Wiener model, having merits in the aspects of interpretability and implementation^25^, and can adapt to the nonlinear fluctuations by cascading the Wiener filter with a polynomial nonlinearity^26^ was further used to develop models with various features selecting criteria. The pre-trained model was further optimized either through the eigenvalues or the periodicities of the possible features to guarantee a set of more promising and efficient decoders. The present work used the Wiener Model for the first time, to the best of our knowledge, to decode the death-related Google search queries that employed death-cause-related Google search queries in Taiwan, resulting in the estimated mortality-related dynamics along with a spectrum of risk factors. The present study systematically explored the relationship between the death-related Google search queries and the death-cause-related Google search queries in Taiwan, aiming to provide new insights into death-related online search behavior at a population level. Our results showed that the death-cause-related search queries are capable of decoding the death-related search queries, indicating the predicting potential of the death-cause-related Google search queries. We expected that this research may provide a basis for the possibility of using Google Trends to predict the upcoming death and causes for the developed island economies in the future. Of note, the present study did not engage with the real-world mortality data directly to the decoding process.

The “Results” and “Discussion” sections compared and discussed the time–frequency analyses of the search queries, the performances of the estimated decoders, as well as the weights of each feature across time lags. The details of the collected datasets, the mathematical details of decoding models, as well as the various decoder’s evaluated measures were introduced in the “Materials and methods” section.

## Results

Nineteen fatal search queries were applied as the candidate predictors to decode two other death-conceptual trends (see Table 1 shows a list of the applied queries and their translation in traditional Chinese), of note, all queries integrated search volumes sampled by weeks lasting for 5 years.

**Table 1:** Information of the 21 search queries, comprising 19 explanatory variables and two estimated variables.

No	Search query	Translation	Variable
1	AIDS	艾滋病	Exp
2	Alzheimer	阿兹海默症	Exp
3	Breast cancer	乳癌	Exp
4	Cancer	癌症	Exp
5	Car accident	車禍	Exp
6	Cirrhosis	肝硬化	Exp
A	Death	死亡	Est
7	Diabetes	糖尿病	Exp
8	Diarrhoeal	腹瀉	Exp
B	Die	死	Est
9	Flu	流感	Exp
10	Heart disease	心臟病	Exp
11	Kidney cancer	腎癌	Exp
12	Lung cancer	肺癌	Exp
13	Malaria	瘧疾	Exp
14	Obstructive pulmonary	阻塞性肺病	Exp
15	Respiratory infection	呼吸道感染	Exp
16	Sick	生病	Exp
17	Stomach cancer	胃癌	Exp
18	Stroke	中風	Exp
19	Tuberculosis	肺結核	Exp

*Exp* explanatory variable, *Est* estimated variable.

**Figure 1:**
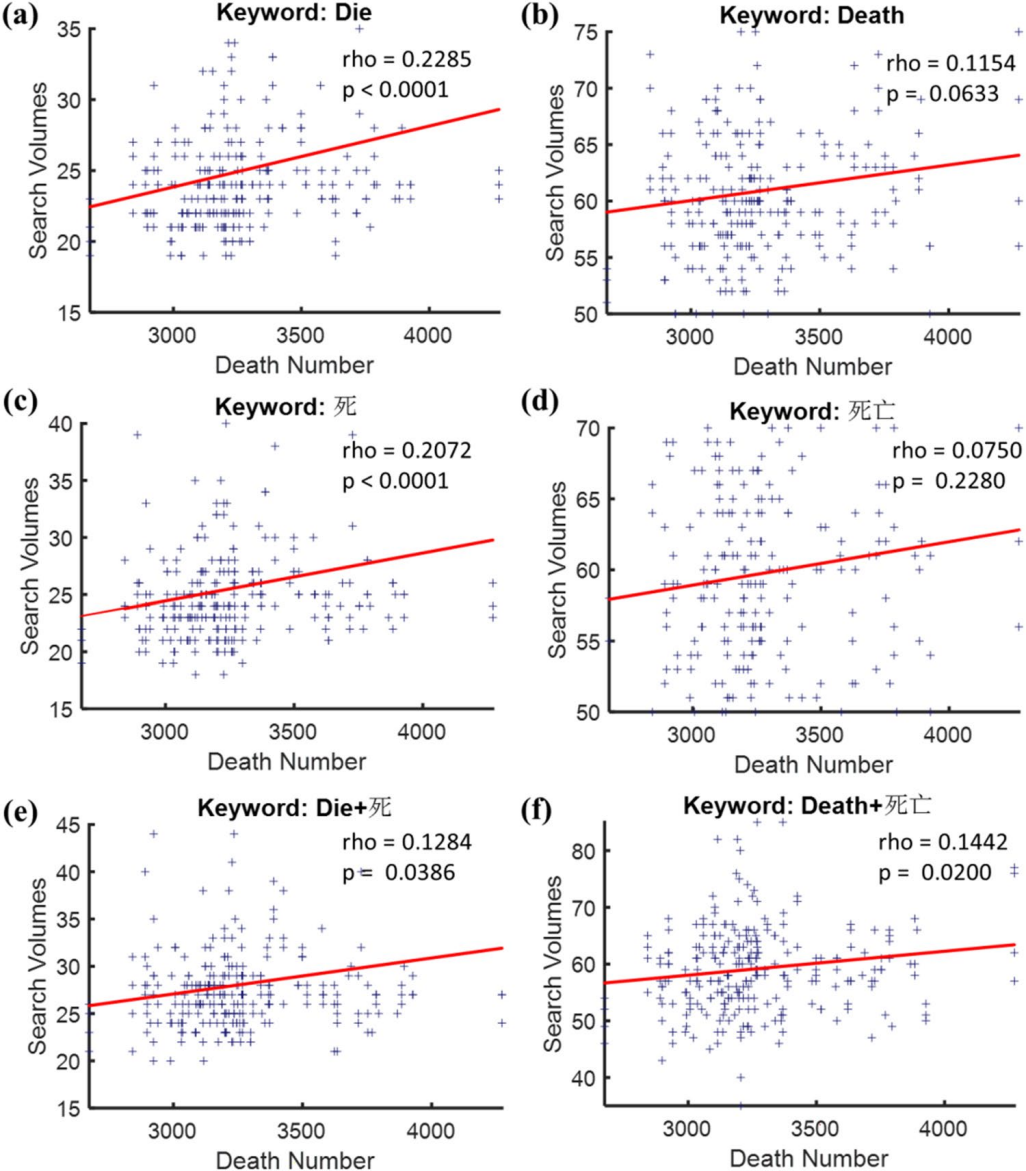
Correlations between the real death number and the search volumes of “Die” (left panels) or “Death” (right panels) using the three different inclusion criteria of search queries. **(a,b)** Include only the English keyword search volumes, while **(c,d)** take the Chinese keyword search volumes as to the source of data. **(e,f)** On the other side, comprise both the English and the Chinese keyword search volumes.

To associate the death-related queries with mortality, correlations between the real death number and the two search volumes (i.e., “die” and “death”) were shown in Fig. 1, wherein different rows of panels compared correlations with search volumes in different languages. The search volumes of the search term “die” were significantly correlated to the real death number across all three sets of keyword search volumes, either in English (Fig. 1a), Chinese (Fig. 1c), or the combined use of the two languages (Fig. 1e); however, no or merely a trend toward significant correlations to the real death number were shown with the term “death” either for the English (Fig. 1b: rho = 0.1154, p = 0.0633) or the Chinese versions (Fig. 1d: rho = 0.0750, p = 0.2280), but still reached a significant correlation with the bilingual version (Fig. 1f: rho = 0.1442, p = 0.0200). In brief, both the terms “Die” (Fig. 1e: rho = 0.1284, p = 0.0386) and “Death” (Fig. 1f: rho = 0.1442, p = 0.0200) presented satisfied and significant correlations to the real death number with the combined use of the two languages. In light of that Taiwan possesses a well-developed Mandarin-English bilingual system and the significant correlations in Fig. 1e,f, bilingual keyword search volumes were applied in the additional analyses.

**Figure 2:**
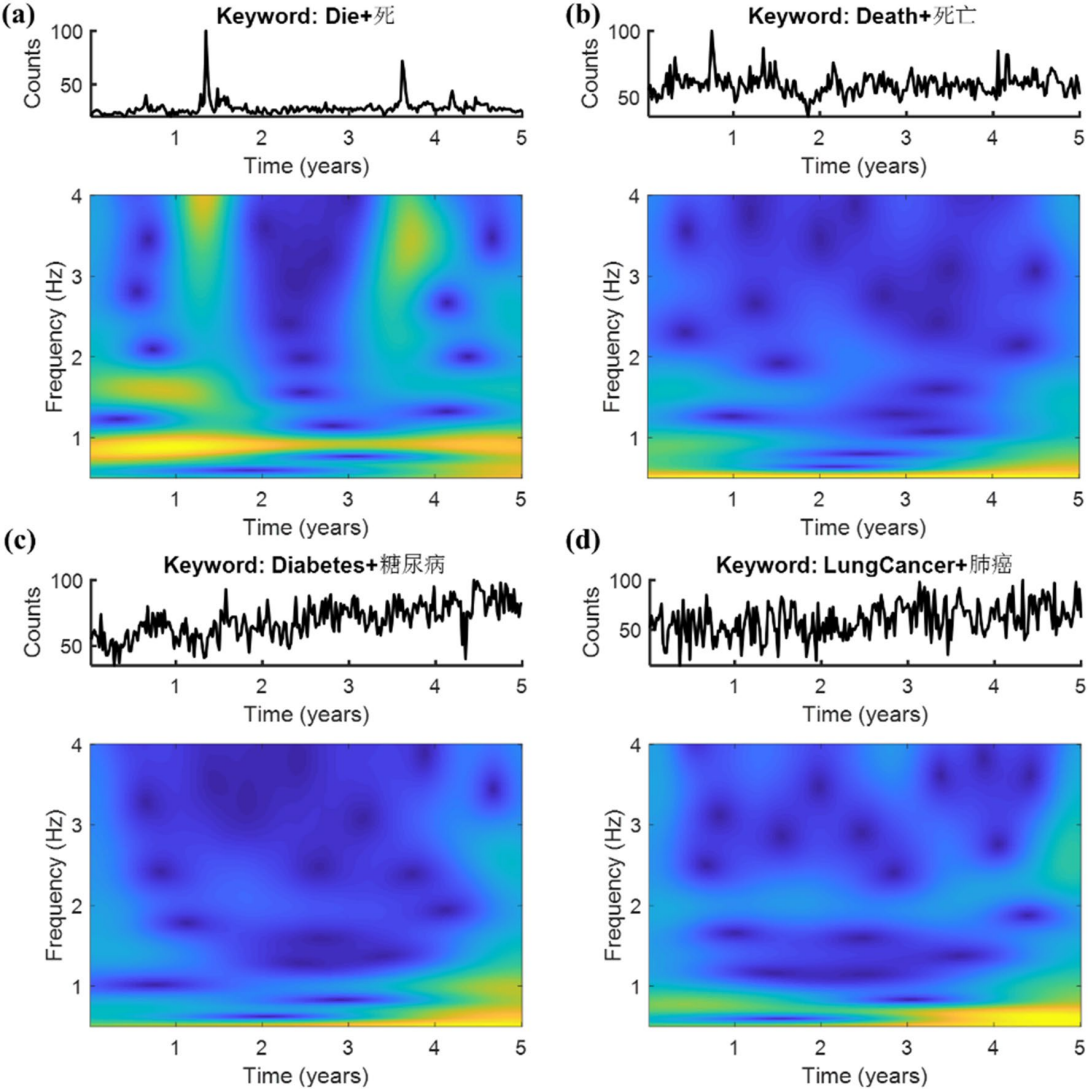
Time series and scalogram of search queries include **(a)** “Die”, **(b)** “Death”, **(c)** “Diabetes”, and **(d)** “Lung Cancer”, all based on the bilingual keyword search volumes.

Figure 2a,b demonstrate time series along with their corresponding scalograms of the two estimated variables include “die” and “death”, respectively; of which the latter possessed both the annual and the semiannual periodic patterns (Fig. 2b), especially the semiannual one; whereas the former (Fig. 2a) presented intermittent annual pattern. On the other side, Fig. 2c shows a representative feature (i.e., diabetes) integrating a relative pure annual periodic oscillation with a monotonic rising trend, while Fig. 2d presents another one (i.e., lung cancer) in which the semiannual periodic pattern dominated the oscillation in contrast. Briefly, a remarkable periodic pattern inferred that the feature was cyclic repetitive either annually, semiannually, or seasonally, thereby might possess a high contribution in predicting the periodic trend. The emergences of periodic patterns of the 21 search queries in bilingual keyword search volumes with visual inspections were summarized in Table 2.

**Table 2:** Periodicities of the 21 search queries in bilingual keyword search volumes.

No	Search query	Variable	Periodicity	Sort
1	AIDS + 艾滋病	Exp	Semi	2
2	Alzheimer + 阿兹海默症	Exp	Semi	3
3	Breast cancer + 乳癌	Exp	Ann	6
4	Cancer + 癌症	Exp	na	19
5	Car accident + 車禍	Exp	na	18
6	Cirrhosis + 肝硬化	Exp	Ann	9
A	Death + 死亡	Est	Semi	na
7	Diabetes + 糖尿病	Exp	Ann	1
8	Diarrhoeal + 腹瀉	Exp	Ann	7
B	Die + 死	Est	Ann	na
9	Flu + 流感	Exp	na	17
10	Heart disease + 心臟病	Exp	Sea	10
11	Kidney cancer + 腎癌	Exp	na	16
12	Lung cancer + 肺癌	Exp	Semi	11
13	Malaria + 瘧疾	Exp	Ann	5
14	Obstructive pulmonary + 阻塞性肺病	Exp	Semi	4
15	Respiratory infection + 呼吸道感染	Exp	Sea	14
16	Sick + 生病	Exp	Ann	12
17	Stomach cancer + 胃癌	Exp	Ann	15
18	Stroke + 中風	Exp	Ann	8
19	Tuberculosis + 肺結核	Exp	Semi	13

*Ann* annual, *Semi* semiannual, *Sea *seasonal, *na* not applicable.

**Figure 3:**
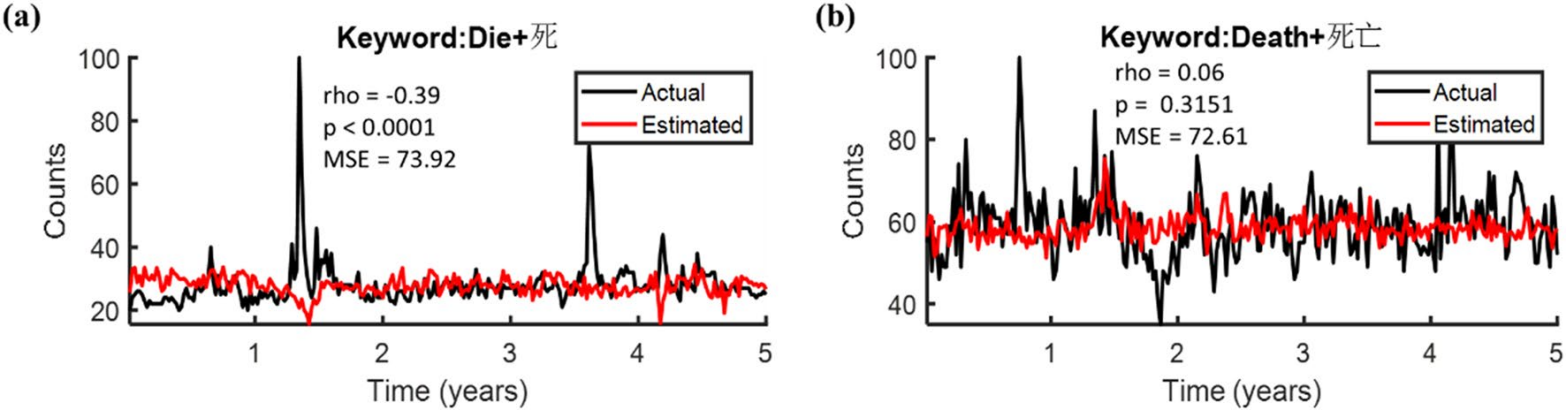
The performances of the multiple linear regression model using all 19 explanatory variables as predictors. Of note, raw bilingual keyword search volumes were applied. Spearman correlations and mean square error between the estimated (red tracks) and the actual (black tracks) trends of the two search terms **(a)** “die” or **(b)** “death” were shown.

To nowcast the death-related dynamics, the multiple linear regression model was first applied to predict the raw bilingual keyword search volumes “die” and “death” from 2015 to 2019 with all 19 explanatory variables as predictors. In Fig. 3a, negative correlation (rho = −0.39, p < 0.0001; MSE = 73.92) was shown between the actual (black track) and the estimated trend (red track) for the search term “die”, inferring the severe phase slips and underestimation. On the other side, as shown in Fig. 3b, a weak positive correlation was revealed for the search term “death” between the real (black) and the decoded trends (red) with its p-value beyond a significant level (rho = 0.06, p = 0.3151; MSE = 72.61).

**Figure 4:**
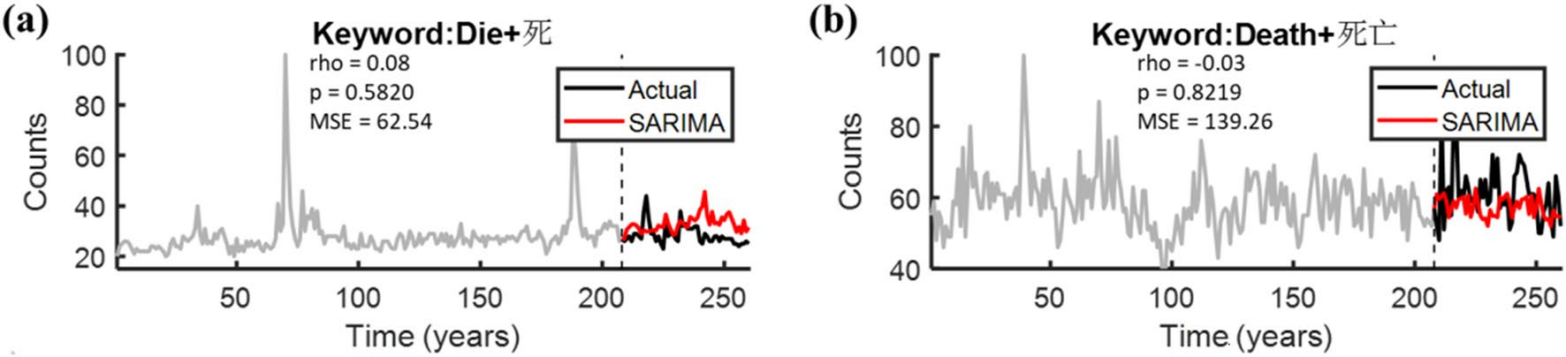
The performances of the SARIMA model. Spearman correlations and mean square error between the SARIMA trends (red tracks) and the actual trends (black tracks) of the two search terms **(a)** “die” or **(b)** “death” were shown.

Next, we implemented the SARIMA model, a well-known autoregressive approach for mortality and/or flu predictions, to estimate the 5th-year trend (2018–2019) in the search volumes of the bilingual queries “die” and “death” based on consecutive fourth year death-related terms per se (2014–2018). In Fig. 4, the red and black tracks correspond to the estimated trends based on the SARIMA model, as well as the actual trends of death-related terms in the 5th year, whilst the gray tracks present the real trends of the death-related queries in the first four years. Unfortunately, neither the search term “die” (Fig. 4a: rho = 0.08, p = 0.5820; MSE = 62.54) nor the term “death” (Fig. 4b: rho = -0.03, p = 0.8219; MSE = 139.26) revealed significant correlations between the SARIMA and the actual trends.

**Figure 5:**
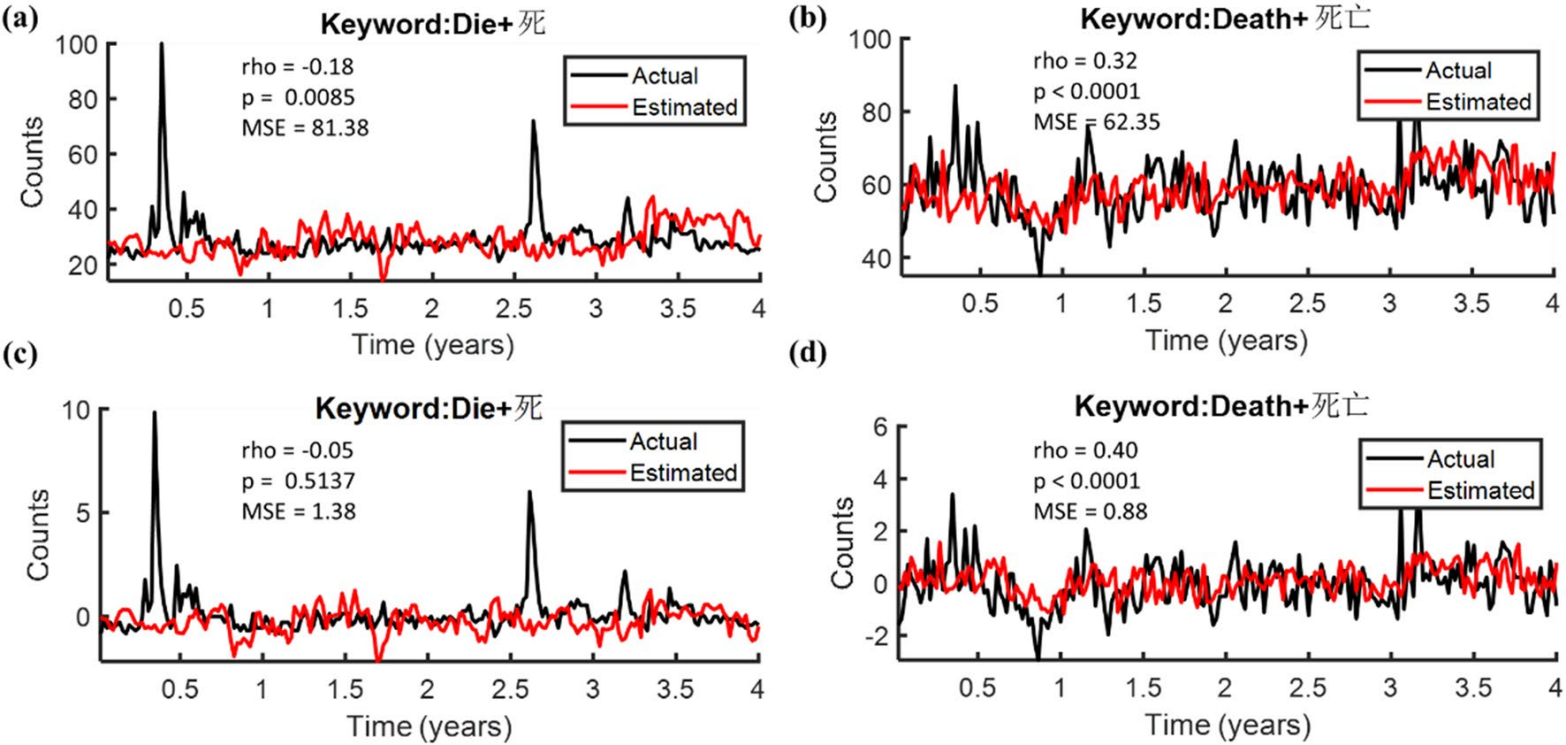
Wiener model decoding performances either without **(a,b)** or with **(c,d)** normalization. The left and right panels correspond to the decoding performances associated with the search terms “die” **(a,c)** and “death” **(b,d)**, respectively.

Next, we introduced the Wiener Model, using all 19 raw bilingual keyword search volumes at time lags up to 52 weeks as the predictors, to decode the search volumes of the death-related queries. The decoding performances with the correlations between the estimated (red tracks) and the actual (black tracks) trends of the search term “die” and “death” were presented in Fig. 5a,b, respectively, wherein the latter showed a significant and positive correlation for the term “death” (rho = 0.32, p < 0.0001; MSE = 62.35), while a negative correlation (rho = -0.18, p = 0.0085; MSE = 81.38) was revealed for the former (i.e., “die”). Considering data may be given by Google in relative terms, a z-score based on normal distribution was employed to the raw search volumes (Fig. 5a,b). With the normalization process (Fig. 5c,d), even higher significant correlation was reached (rho = 0.40, p < 0.0001; MSE = 0.88) for the search term “death” (Fig. 5d), as compared to that without (Fig. 5b). Our results supported the z-score process as a useful technique to eliminate the potential uncertainty in relative quantities as well as to improve the decoding performances with the Wiener Model. Unfortunately, it failed to reach a significant correlation for the search term “die”.

Comparisons of decoding performances among the three different methods (Wiener model, Multiple Linear Regression, and SARIMA) either with or without normalization were summarized in Table 3. The Wiener model showed the best fit for the data, especially for the search term “death”. With normalization to the time series, the Wiener model was the only method that presented significant positive correlations between the estimated and the actual trends for the term “death” (rho = 0.40, p < 0.0001). Similarly, the Wiener model also outperformed the rest two approaches (Multiple Linear Regression, and SARIMA) with the direct use of the raw search volumes, revealing positive correlations to the actual trends with the term “death” (rho = 0.32, p < 0.0001). Of note, the decoding performances with normalization were superior to those without in general.

**Table 3:** Decoding performances among the three different methods either with or without normalization.

Normalization	Method	Estimated variable	rho	p-value	MSE
No	Wiener model	Die + 死	−0.18	**0.0085**	81.38
		Death + 死亡	0.32	** < 0.0001**	62.35
	Multiple linear regression	Die + 死	−0.39	** < 0.0001**	73.92
		Death + 死亡	0.06	0.3151	72.61
	SARIMA	Die + 死	0.08	0.5820	62.54
		Death + 死亡	−0.03	0.8219	139.26
Yes	Wiener model	Die + 死	−0.05	0.5137	1.38
		Death + 死亡	0.40	** < 0.0001**	0.88
	Multiple linear regression	Die + 死	−0.38	** < 0.0001**	1.38
		Death + 死亡	0.06	0.3183	1.08
	SARIMA	Die + 死	0.08	0.5820	1.17
		Death + 死亡	−0.03	0.8219	2.08

Bold fonts indicate p-value < 0.05.

**Figure 6:**
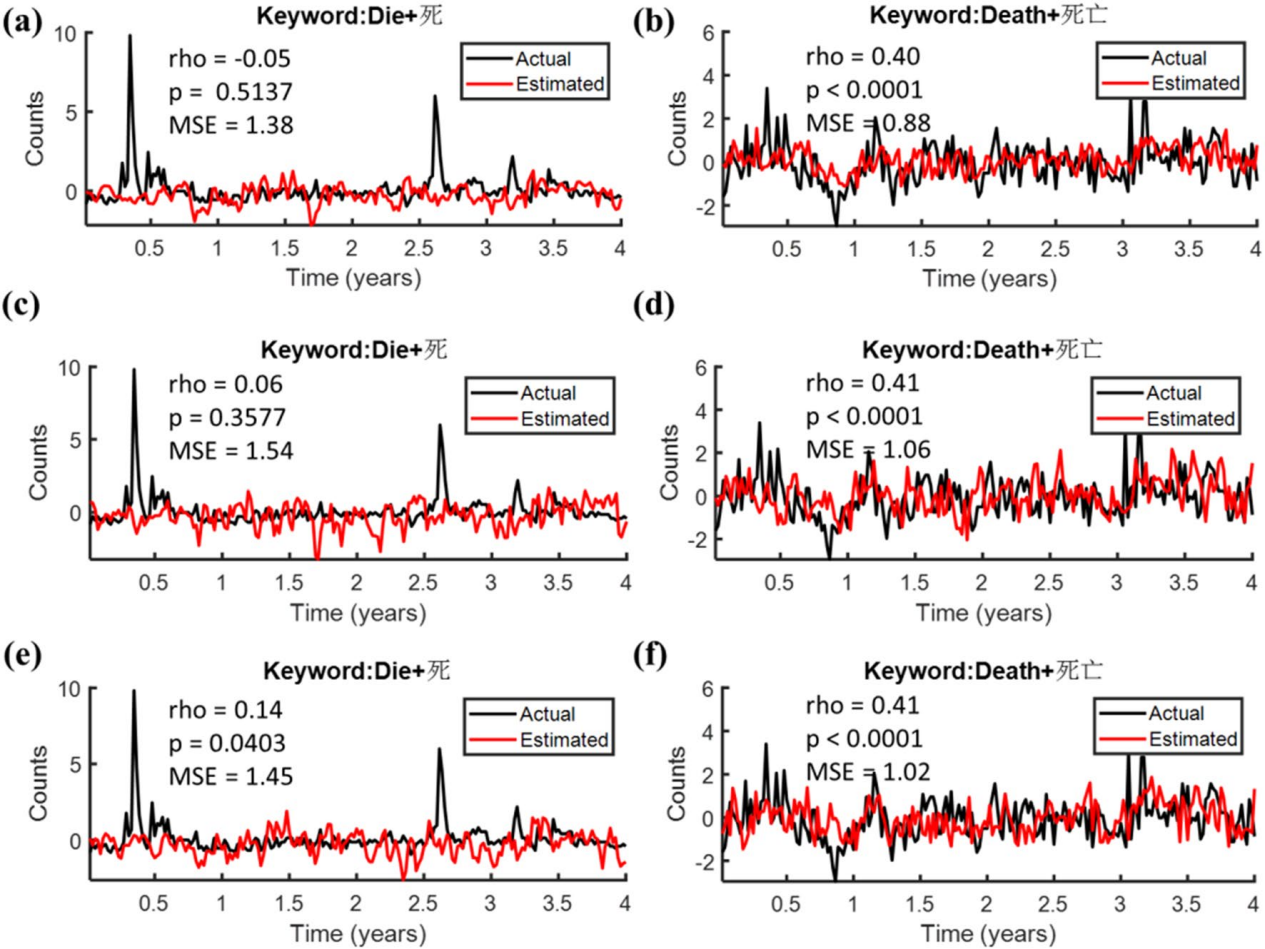
Wiener model decoding performances under the three different features selecting strategies. All bilingual keyword search volumes were normalized. Spearman correlations and mean square error between the estimated (red tracks) and the actual (black tracks) trends of the two search terms “die” (left panels) or “death” (right panels) were presented. **(a,b)** The estimated trends with all possible features included. Two other feature selection criteria were applied to optimize the models. **(c,d)** The performances with the ten remarkable periodic features. **(e,f)** The performances with the ten highest-weight features according to **(a**,**b)**.

Given the superiority of the Wiener model, two other feature selection strategies were proposed to optimize the models. Figure 6 shows the comparisons of decoding performances among the three different feature selection strategies based on the Wiener model. Figure 6a,b nowcast the search term “die” and “death” with all 19 explanatory variables in Table 1 set as the predictors, revealing significant correlations between the actual (black tracks) and the estimated trends (red tracks) for the search term “death” (Fig. 6b: rho = 0.40, p < 0.0001; MSE = 0.88); nevertheless, it failed to present a significant correlation for the search term “die” (Fig. 6a: rho = −0.05, p = 0.5137; MSE = 1.38). Next, we considered periodicities of all candidate predictors as the feature selection criterion, whilst including the ten features revealing remarkable periodic patterns, referenced to time series and scalogram of each search query (Fig. 2 and Table 2). Similar to the performances with all possible feature included, the correlation between the estimated (red tracks) and the actual (black tracks) trends for the search term “die” (Fig. 6c) was slightly improved (rho = 0.06, p = 0.3577; MSE = 1.54) compared to that with all features included (Fig. 6a), but still failed to reach a significant level; whilst the term “death” (Fig. 6d) maintained a comparable performance (rho = 0.41, p < 0.0001; MSE = 1.06) to that with all possible features (Fig. 6b). These results may suggest that periodicity is a feasible feature selection criterion.

**Figure 7:**
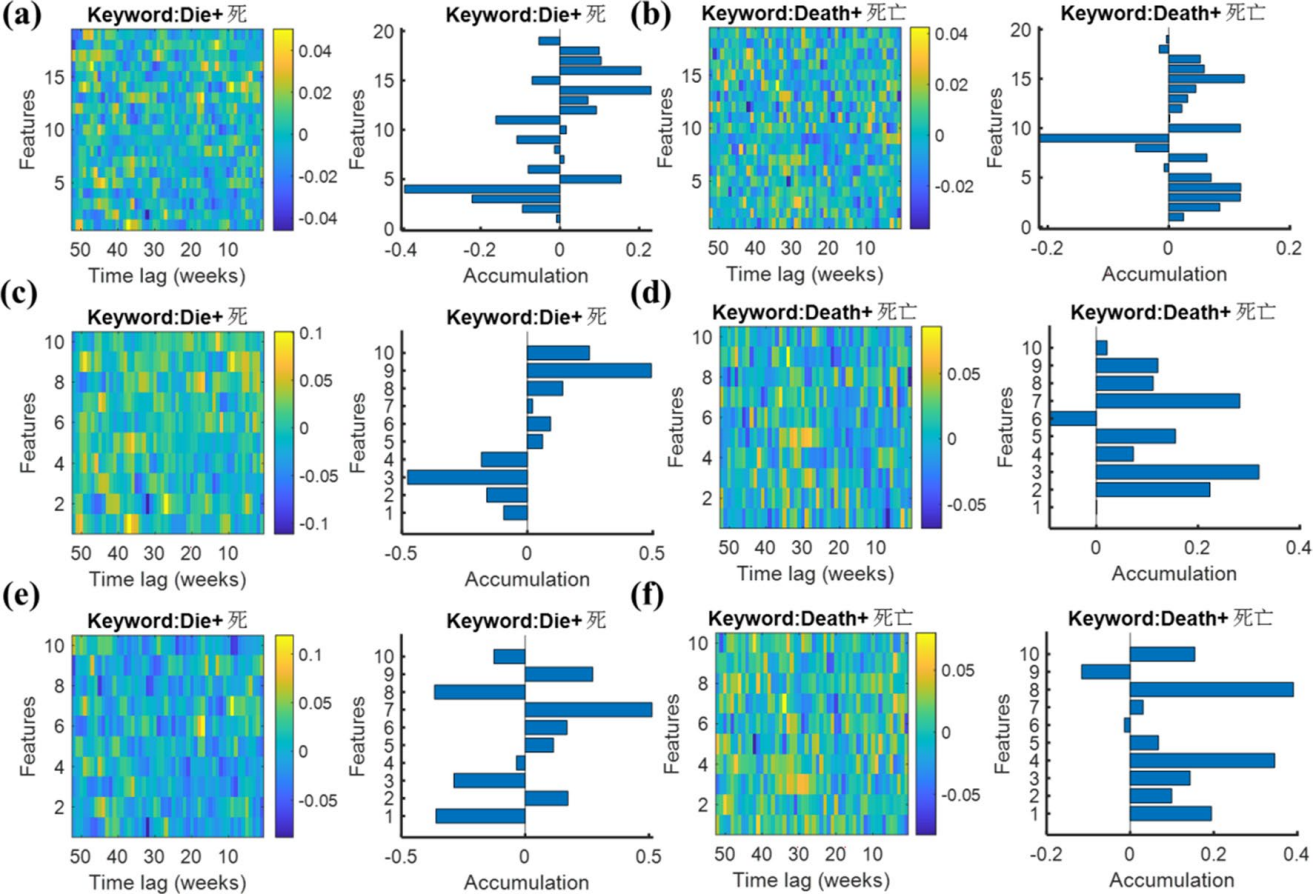
Features importance to decode the death-related terms with normalization in search queries. **(a,b)** The weight distribution across all time lags and the integrated weight of all features for the search term “die” and “death”, respectively. Two other feature selection criteria were applied to optimize the models. **(c,d)** The results with the ten most remarkable periodic features. **(e,f)** The performances with ten highest-weight features from **(a,b)**.

The color plots (left panel for each subplot) in Fig. 7 present the contributions of all candidate features, at time lags up to 52 weeks with a step in one week, to decode the five-year long-term death-related search terms. To compare and sort the contribution of each feature, the weight distribution of each candidate feature were integrated across all time lags, generating a bar plot (right panel for each subplot in Fig. 7) which shows the sum of weights of each feature; thereby the ranks of all candidate features in Table 1 could be determined by sorting this sum of weights with all possible features as the predictors for the search terms “die” (Fig. 7a) and “death” (Fig. 7b), respectively. Table 4 displays the weight orders of the 19 explanatory variables for the two death-related queries “die” or “death”. Surprisingly, the correlation between the estimated (red tracks) and the actual (black tracks) trends for the search term “die” (Fig. 6e) was significantly improved (rho = 0.14, p = 0.0403; MSE = 1.45) compared to the other two feature selection criteria (Fig. 6a,c). Both the term “die” (Fig. 6e: rho = 0.14, p = 0.0403; MSE = 1.45) and the term “death” (Fig. 6f: rho = 0.41, p < 0.0001; MSE = 1.02) presented significant positive correlations, implying the sum of feature weights as a reliable criterion in minimizing the number of selected features, whilst reaching a satisfactory decoding performances.

**Table 4:** Weight orders of the 19 explanatory variables for the two death-related queries “die” or “death”.

No	Search query	W_die_	W_death_
1	AIDS + 艾滋病	10	12
2	Alzheimer + 阿兹海默症	15	5
3	Breast cancer + 乳癌	18	4
4	Cancer + 癌症	19	3
5	Car accident + 車禍	3	6
6	Cirrhosis + 肝硬化	14	16
7	Diabetes + 糖尿病	9	7
8	Diarrhoeal + 腹瀉	11	18
9	Flu + 流感	16	19
10	Heart disease + 心臟病	8	2
11	Kidney cancer + 腎癌	17	14
12	Lung cancer + 肺癌	6	13
13	Malaria + 瘧疾	7	11
14	Obstructive pulmonary + 阻塞性肺病	1	10
15	Respiratory infection + 呼吸道感染	13	1
16	Sick + 生病	2	8
17	Stomach cancer + 胃癌	4	9
18	Stroke + 中風	5	17
19	Tuberculosis + 肺結核	12	15

The ten highest-weight features, as reported in Table 4, included “Alzheimer”, “Car Accident”, “Diabetes”, “Heart Disease”, “Lung Cancer”, “Malaria”, “Obstructive Pulmonary Disease”, “Respiratory Infection”, “Sick”, and “Stomach Cancer” (No. 1 to No. 10 in Fig. 7e,f); whereas the ten features possessed the most remarkable periodic patterns, as reported in Table 2, included “AIDS”, “Alzheimer”, “Breast Cancer”, “Cirrhosis”, “Diabetes” (Fig. 2c), “Diarrhoeal”, “Heart Disease”, “Malaria”, “Obstructive Pulmonary Disease”, and “Stroke” (No. 1 to No. 10 in Fig. 7c,d), which mainly characterized by either annual, semiannual or seasonal periodic patterns. As shown in Fig. 7c, the three features showing the highest sum of weights to forecast “die” were “Obstructive Pulmonary Disease”, “Stroke”, and “Malaria” in order; in contrast, “Breast Cancer”, “Heart Disease” and “Alzheimer” were the top three to decode the trend for “death” (Fig. 7d). The ten candidate features for Fig. 7e,f were determined by the sorted sum of weights from Fig. 7a,b. With the ten highest-weight features’ selecting strategy, the three most contributed features in predicting the search term “die” were “Obstructive Pulmonary Disease”, “Sick”, and “Car Accident” (Fig. 7e), while “Respiratory Infection”, “Heart Disease”, and “Alzheimer” contributed more to the search term “death” (Fig. 7f). Table 5 summarizes all statistical results (i.e., MSE, and rho along with its p-value) in predicting the death-related terms (i.e., “die” or “death”) with predictors determined by the three different feature selection criteria (i.e., all features, periodicity, and weight), either with or without normalization process. Briefly, a z-score prior to the Wiener Model improved the decoding performance overall, whilst both feature selection strategies including “ten most periodic features” and “ten highest-weight features” presented comparable performances to that with all possible features for the term “death” in general. With the normalization process, selecting predictors according to the weight orders of the 19 explanatory variables outperformed that based on the oscillatory periodicity for the term “die”, whereas the features selecting strategy with oscillatory periodicity favored the nowcast for the term “death”.

**Table 5:** Comparisons of performances (rho with its p-value, and MSE) in decoding the estimated variables (i.e., “Die” and “Death” in bilingual keyword search queries) with the three types of features selecting strategies (i.e., all possible features, the ten most periodic features, and the ten highest-weight features).

Normalization	Selection	Estimated variable	rho	p-value	MSE
No	All possible features	Die + 死	−0.18	**0.0085**	81.38
		Death + 死亡	0.32	** < 0.0001**	62.35
	Ten most periodic features	Die + 死	−0.04	0.5716	79.99
		Death + 死亡	0.36	** < 0.0001**	76.62
	Ten highest-weight features	Die + 死	0.12	0.0921	87.25
		Death + 死亡	0.37	** < 0.0001**	82.17
Yes	All possible features	Die + 死	−0.05	0.5137	1.38
		Death + 死亡	0.40	** < 0.0001**	0.88
	Ten most periodic features	Die + 死	0.06	0.3577	1.54
		Death + 死亡	0.41	** < 0.0001**	1.06
	Ten highest-weight features	Die + 死	0.14	**0.0403**	1.45
		Death + 死亡	0.41	** < 0.0001**	1.02

Bold fonts indicate p-value < 0.05.

## Discussion

### The usefulness of search query

Search engine query data such as Google Trends has been applied as a potential data source to detect influenza activities. For example, Ginsberg et al. selected 45 search queries data to detect influenza-like illness activity in the United States^7^. Later, Araz et al. showed that the additional use of Google Trends search query data improved the performance of the linear regression models by comparing the root means square errors (RMSEs)^10^. Recently, Mavragani et al. used search query data from Google Trends, forecasting AIDS prevalence in the United States with the AIDS-related search terms, which supported the conclusion of past findings that Google Trends data are valid and valuable for the analysis and forecasting of human behavior towards health topics^17^. In another study, Lu et al. predicted the occurrence of epidemic avian influenza using Google Trends data with the multiple linear regression model, indicating a hybrid set of predictors containing information from Google Trends will be a plus^27^. This paper attempts to show the relationship between the death-cause-related search queries and the death-related search queries (i.e., “die” and “death”), providing a deeper insight into users’ online search behavior about mortality at a population level. Whilst this study did not validate the real-world mortality decoder, it did partially examine the feasibility and reliability of the use of Google Trends in predicting mortality, by checking if the search query data resemble that of the official mortality records. Our results, as shown in Fig. 1, proved that the bilingual death-related search query volumes obtained from Google Trends (i.e., “die” and “death”) significantly correlated to the real death number, prevailing across the English and Chinese search queries, thus supporting the search query data from Googles might have potential in predicting the real mortality.

### Candidate keywords selection

To effectively predict the possible death, we first included candidate features according to the main death causes in Taiwan, referenced to the common death causes reported by the World Health Organization. Sixteens out of nineteen potential death cause included in this work were determined by this criterion. On the other side, four out of nineteen candidate features (i.e., cancer, car accident, flu, and sick) were determined based on the general habitual uses in Google searching (e.g., “sick”) or the extended concepts of the former sixteen features (e.g., “cancer”). Criteria for selecting these four death causes were lied on several general habitual uses in Google searching that may potentially link to the possible death, e.g., “car accident” is well-known as one of the critical causes of death in Taiwan, “flu” may serve as an antecedent or contributory cause of death, while “sick” and “cancer” are the more general frequent usages to show the illness thus might be useful in prediction. Of note, these 19 features may either be categorized as the immediate, the antecedent, or the contributory causes of death.

### Search query occurrence

The periodic pattern of certain Google search queries (Table 2) may be associated with the seasonal effects of these diseases. For example, some diseases are directly influenced by seasons, such as malaria^28^, cirrhosis^29^, diarrhoeal^30^, stroke^31^, and heart disease^32^. Other diseases, although may not be season-driven diseases per se, presents seasonal patterns for various reasons. For example, breast cancer incidence has seasonal patterns that seem to vary among global populations^33^. The associated symptoms of diabetes are influenced by seasonality, as reported in the past study that the Gestational Diabetes Mellitus prevalence in Taiwan revealed seasonal variation, with the highest risk occurring in spring and summer due to the post-glucose load level variations^34^, thus may also contribute to the periodicity of the online search volumes. It has also been reported that season has a clinically significant influence over the cognition function in older adults either with or without Alzheimer’s diseases, suggesting the associated symptoms of Alzheimer’s diseases are more likely to be pronounced in the winter and early spring^35^. The discussions mentioned above may provide a possible explanation for the emerging periodicity of the search query, however, the exact reason underlying these trends required further validation with the specific death-cause-related mortality records.

### Decoding performances of the three models

In comparisons of the mortality-related decoding performances between the three potential models (i.e., Wiener model, Multiple Linear Regression, and SARIMA), the Wiener Model outperformed the other two in general, as shown in Table 3, .e., the estimated search queries “death” presented positive significant correlations to the actual trends, especially prominent for those with normalization. Given the mechanism of the multiple linear regression model, a possible reason for the unsatisfied decoding performances might be the insufficient number of predictors, and the lack of an effective optimization process. On the other side, the predicting performances for the SARIMA models failed to fit the actual trends (see Fig. 4), which may be resulted from the nonlinear nature of the pattern, ambiguous seasonality of the data, as well as the insufficient length of the inputs for the autoregressive analysis. Of note, the SARIMA model requires preliminary analysis to determine the parameters of the model, thus complicating the analysis. The Wiener Model, an extension to the traditional multiple linear regression model, adopts different features at various time lags as the predictors. The implementation of the Wiener–Hopf equation to optimize the corresponding weights, along with a ridge regression to overcome the overfitting problem, all support the Weiner model presented more accurate estimates than the traditional linear regression model. Meanwhile, the Wiener–Hopf equation further facilitates and benefits the computation efficiency for the weights of features as well, plus the availability for the Wiener Model in dealing with the nonlinear oscillations, both support its superiority over that of the SARIMA model.

### Importance of normalization process

Considering Google Trends normalizes data by the total search volumes over a scale from 0 to 100, then represents and visualizes as the weekly relative search volumes, it is a necessity to correct results for population size and makes it fair to compare data across different keywords. Taking into account the baseline for estimation varies over time, a standard transformation z-score method was employed to normalize the relative search volumes from Googles based on the normal distribution.

The algorithm of z-score normalization is formulated as *z* = *(x − u)/σ*, where *u* is the mean and *σ* denotes the standard deviation of the input. Our results showed that the z-score normalization eliminated the varying baseline of search volumes across different queries, improving the decoding performances overall with Wiener models (Table 3), with its benefits prevailing across all three different feature selecting strategies (Table 5), both manifesting the importance of normalization in processing the data from Google Trends.

### Feature selecting strategies

The last concern of this work goes to the timing of the use with the different feature selecting strategies. Our results, as summarized in Table 5, indicated either periodicities or weights of predictors were critical for the estimations, manifesting certain overlaps may occur between the selecting strategies with the ten most periodic features and the ten highest-weight features (five out of ten), i.e., partial periodic alternating search queries shared high weights thus could serve as the general predictors in practical use, including “Alzheimer”, “Diabetes”, “Heart Disease”, “Malaria”, and “Obstructive Pulmonary Disease” (see Table 2, Table 4, and Fig. 7). As for those high-weights but non-periodic features, including “Car Accident”, “Lung Cancer”, “Respiratory Infection” “Sick” and “Stomach Cancer”, tended to contribute more to the death-related terms with multiple competing oscillatory components (e.g., “die”) than that dominated by the annual or semiannual periodic patterns (e.g., “death”).

The search query “die” may associate with a fear-driven fatal disease outbreak, such as the abrupt changes due to an emergent occurred event or those with annual prevalence, as shown in Fig. 2a. Following this, only the feature selecting strategies with the ten highest-weight features presented significant positive correlations compared to that with the ten most periodic features selecting strategies (Table 5). In contrast, the search query “death” presented the overall trend that comprises the multiscale oscillatory patterns corresponding to the multiple death causes with less contamination by the emergent occurred event (Fig. 2b). Therefore, both the feature selecting strategies with the ten most periodic features or with the ten highest-weight features presented similar and significant correlations and were slightly higher for the latter (Table 5).

## Conclusion

This study explored the death-related online search behavior in Taiwan based on three different methods with the engagement of a set of death-cause-based search queries from Google Trends. Our results showed the Wiener models outperformed the multiple linear regression model and the SARIMA in terms of the correlations between the estimated and the actual trends for the death-related queries. Of note, significant correlations between the bilingual death-related queries and the authentic death number in Taiwan were validated. Moreover, both the feature possessed remarkable periodicity and of high-weight contributed to similar performances for the term “death”, while only the high-weighted features favored the term “die” and presented significant correlations.

## Materials and methods

### Dataset collection and feature extraction

Keyword search volumes from Google Trends (https://Trends.google.com/Trends) were assessed as our datasets. Table 1 summarizes 21 variables, among them nineteens as the explanatory variables (i.e., No. 1 to No. 19), whereas the rest two are the estimated ones (i.e., No. A and No. B). The two death-related estimated variables include “die” and “death”. All search volumes of the 21 variables were automatically normalized by Google Trends, ranging from 0 to 100. The explored features were determined by the main death causes in Taiwan, or their extended concepts. Among the nineteen potential death causes, sixteens were referenced to the common death causes reported by the World Health Organization, except for the four extended terminologies (i.e., cancer, car accident, flu, and sick). These nineteen potential death-related factors may either serve as the immediate, antecedent, or contributory causes of death. The immediate death cause defines as the reasons which directly cause the death, whereas the antecedent death cause refers to the underlying diseases that causally lead to the immediate cause. The contributory death cause facilitates the death (e.g., diabetes), however not necessarily relates to the immediate and/or the antecedent causes. All search queries spanned from Oct. 2014 to Sep. 2019. Of note, the data that support the findings of this study are available from the corresponding author upon reasonable request. A sliding and non-overlapping window with a fixed length in a week was applied (i.e., 52 counts/year), to reach a moderate resolution and/or oscillatory variation of a time series.

The monthly official mortality data were collected from the National Statistics Network in Taiwan (https://www1.stat.gov.tw), covering all ages and all death causes. As aforementioned, the search volumes from Google Trends were sampled by weeks while the official mortality data were presented monthly; thus, to match and align the samples for an approximate correlation between the Google search volumes and the real mortality records, the monthly death number was split evenly by weeks, wherein the death number of the week covering adjacent months was weighted by the lasting days of each. Spearman correlation analysis was then applied to validate that the incidence of the words “death” and “die” resembled that of the real mortality records. To guarantee a satisfactory performance whilst minimizing the number of features applied, a finer sorting algorithm may fulfill the needs. To this end, a complex Morlet Wavelet transform was first applied to generate a time–frequency representation of each weekly search query (e.g., Fig. 2). Next, these nineteen predictive features were categorized and sorted according to either the periodicities or the eigenvalues of each explanatory variable. The selections of features are rooted on three types of criteria including all search terms as features, or features with cyclic alternating patterns determined by the periodicities of search queries according to their Wavelet scalograms, or involving only the high-weighted features based on the decoding performances with all possible features set as predictors. All methods were carried out in accordance with relevant guidelines and regulations.

### Multiple linear regression model

All candidate explanatory variables were used as predictors to estimate the search volume of “die” or “death” from 2015 to 2019. The linear regression model is formulated as ${y}_{t}={\beta }_{0}+ \sum_{i=1}^{19}{\beta }_{i}{x}_{it}$, where ${\mathrm{y}}_{\mathrm{t}}$ represents the estimated mortality at time *t*, ${x}_{it}$ refers to the *i*th predictor at the same time point *t*, while ${\beta }_{i}$ stands for the linear regression coefficients.

### SARIMA model

The Seasonal Autoregressive Integrated Moving Average (SARIMA), an extension to the Autoregressive Integrated Moving Average (ARIMA) model, supports the modeling with the seasonal modulation in the series. The general form of the SARIMA model can be expressed as ARIMA (*p, d, q*) × (*P, D, Q*) *S*, where *p*, *d*, and *q* corresponds to the number of the autoregressive terms, the non-seasonal differences, and the moving-average terms, respectively; *P*, *D*, and *Q* stands for the number of the seasonal autoregressive terms, the seasonal differences, and the seasonal moving-average terms, while S denotes the periodic terms.

In this work, we constructed an ARIMA (1,1,1)$\times$(0,1,1) model to estimate the continuation of the search volumes of “die” and “death” respectively. Considering that the estimated variables “death” possessed both the annual and the semiannual periodic patterns (Fig. 2b), especially for the semiannual one; While the term “die” presented the intermittent annual pattern (Fig. 2a). Hence, the SARIMA model implemented in this work was embedded with a periodicity of 52 weeks (S = 52) for “die”, and with a periodicity of 26 weeks (S = 26) for “death”, of note, both were performed with the data from 2014 to 2018 as the training set, and the data from 2018 to 2019 as the test set.

### Wiener filtering and cascade model

The Wiener filter^25^, which is similar to that of the multiple linear regression, aims to seek the weights of a weighted sum of different features. In this work, the estimated mortality was set as the dependent variable, while the search queries at various time lags were set as the explanatory variables. The linear representation of a Wiener filter is formulated as $\overline{M }\left(t\right)=\sum_{j=0}^{Q}\sum_{i=1}^{P}{a}_{ij}{x}_{i}(t-j)$, where $\overline{M }\left(t\right)$ represents the estimated mortality at time *t*, ${a}_{ij}$ stands for the weight corresponds to the *i*th feature (*P* features in total) at a time lag of *j* (*Q* time lags in total), whereas ${x}_{i}(t-j)$ refers to the *i*th feature at a time lag of *j*.

To estimate and optimize the corresponding weights, the Wiener–Hopf Equation^25^, a set of linear equations formulated as $\mathrm{A}={({X}^{T}X)}^{-1}{X}^{T}M$, minimizes the sum of the squares of the difference between the estimated mortality $\overline{M }\left(t\right)$ and the actual death-related search query $M(t)$, has the inside track for its computational efficiency with a closed-form solution, where *A* is a $P(Q+1)\times 1$ matrix of ${a}_{ij}$, *X* represents all features at various lags (maximum time lags = 52 weeks) with a $N\times P(Q+1)$ matrix, N stands for the number of sliding windows, whereas M denotes the actual death-related queries with $\mathrm{N}\times 1$ vector. Next, a ridge regression *A*^2^ was added to the Wiener–Hopf equation as a regularization term to deal with overfitting, which is formulated as $\mathrm{A}={({X}^{T}X+\lambda I)}^{-1}{X}^{T}M$, where λ denotes the regularization parameter^26^.

To go a step further, a Wiener Cascade Model^26^ was built by cascading the output of the Wiener filter to a static nonlinear unit, to model nonlinear relationships between the predictors and the estimated trends. In this work, a 3rd order polynomial unit was applied with the corresponding weights to be estimated by the least-squares approach.

### Decoder evaluations and statistical analyses

Before the decoding process, the z-score method was applied to normalize the raw time series. The decoding performances of the estimated trends were first evaluated by calculating the Spearman’s rank correlation coefficient (rho), a non-parametric measure of correlation without the need to fulfill any assumptions in the frequency distribution of the inputs, to measure how well a monotonic function represents the relationship between the estimated and the actual trends of the death-related series, of note α = 0.05 was set for all hypothesis testing. On the other side, the mean square error (MSE) was also used to access the quantitative differences between the estimated and the actual trends, thereby ensuring the oscillatory differences were refined within a sufficiently small error. In this work, we applied the fivefold cross-validation algorithm to train the multiple linear regression model and the Wiener Model, of which 4 of the 5 folds were served as the training datasets to test the other one fold, respectively. For the Wiener Model, similar decoder evaluations and statistical analyses were applied to models with different sets of included features. All analyses were conducted using MATLAB (MathWorks, Natick, MA).

### Limitations of the study

One important limitation of nowcasting mortality-related concepts using data from Google Trends must be borne in mind is that the database is oriented from the time-evolving search volumes of queries, thus specific discussions, such as critical features in predicting mortality, which may change over time, require additional validation. In addition, to secure the relative homogeneity of the dataset as well as to the pursuit of the nowcast based on the one-year time lags, we only included the one-year historical data into the prediction. For those with longer time lags which could be relatively diverse in patterns is beyond our scope. On the other side, to correlate the Google search volumes with the official mortality records, we assumed that the monthly death number follows weekly uniform distributions, resulting in an approximation but not a precise estimation. Another point that was not validated yet is the linkages between the death-cause-related search volumes and the clinical diagnostic records showing the disease prevalence in the population, as we aimed to examine the effectiveness of the death-cause-related search queries in prediction, exploring such linkages is beyond our scope. The last limitation is that the present study did not engage with the real-world mortality records in developing predicting models, as the interest of the present work lies in exploring the degree to which death-cause-related online behavior could predict the death-related online behavior at a population level, as well as to offer potential mortality-related decoders along with guidance to the critical predictors in death-cause-related search queries, thus such analyses are beyond our scope (see Suppl. Information for full name and description for abbreviation).

## Supplementary Information

Supplementary Table S1.
