# Supplementary material for: Query-based-learning mortality-related decoders for the developed island economy
Source: Sci Rep. 2022 Jan 19;12:956. doi: 10.1038/s41598-022-04855-2 (PMC8770507; doi:10.1038/s41598-022-04855-2)
Supplement: Supplementary file 1 — Supplementary Table S1. [file 41598_2022_4855_MOESM1_ESM.docx]

**Table S1.** Full name and description for abbreviation

| **No.** | **Abbreviation** | **Full Name** | **Description** |
| --- | --- | --- | --- |
| 1 | ARIMA | Autoregressive Integrated Moving Average | An autoregressive model for time series analysis. |
| 2 | SARIMA | Seasonal Autoregressive Integrated Moving Average | An extension to the ARIMA model that supports seasonal modulation. |
| 3 | MSE | Mean Square Error | The average of the squares of the errors. |
| 4 | RMSE | Root Mean Square Error | The differences between estimated and actual values. |
| 5 | AIDS | Acquired Immunodeficiency Syndrome | A disease spectrum of human immune system caused by infection with human immunodeficiency virus. |
| 6 | exp | explanatory variable | Variable as predictors to decode the estimated variable. |
| 7 | est | estimated variable | Variable to be estimated. |
| 8 | ann | annual | Annual periodic pattern. |
| 9 | semi | semiannual | Semiannual periodic pattern. |
| 10 | sea | seasonal | Seasonal periodic pattern. |
| 11 | na | not applicable | None of the selections applied. |
